# Facile Synthesis, Characterization, and *In Vitro* Antimicrobial and Anticancer Activities of Biscoumarin Copolyester Bearing Pendant 3-(Trifluoromethyl)Styrene

**DOI:** 10.1155/2014/369473

**Published:** 2014-10-28

**Authors:** Narendran Kandaswamy, Nanthini Raveendiran

**Affiliations:** Postgraduate and Research Department of Chemistry, Pachaiyappa's College, Chennai 600 030, India

## Abstract

Synthesis of random biscoumarin copolyester bearing pendant 3-(trifluoromethyl)styrene was prepared by the reaction of biscoumarin monomer **3** and hydroquinone **5** with azeloyl chloride. The influence of pendant 3-(trifluoromethyl)styrene unit on the properties of copolyester such as inherent viscosity, solubility, and thermal stability was investigated and compared in detail. The inherent viscosity and polydispersity index of the copolyester were found to be 0.15 dL/g and 1.36, respectively. The chemical structure of the copolyester was investigated by Fourier-transform infrared spectroscopy (FTIR) and proton nuclear magnetic resonance (^1^H-NMR) spectroscopy. The physical properties of copolyester were characterized by thermogravimetric analysis (TGA), differential scanning calorimetry (DSC), gel permeation chromatography (GPC), and X-ray diffraction (XRD) technique. Agar disc diffusion method was employed to study the antimicrobial activity of the random copolyester. *In vitro* anticancer activity against lung cancer (*Hep-2*) cell line was also investigated.

## 1. Introduction

Aliphatic aromatic copolyester [1–3] bears high thermal stability and excellent mechanical strength and is being used in various disciplines. A probe through the literature indicates that there has been a rising interest in synthesizing aliphatic aromatic copolyester [4–6] as biomaterials [7] for a variety of biomedical applications. In spite of unique properties such as thermal stability, good chemical resistance, and excellent mechanical strength, these polymers encounter the problem of fabrication process due to high glass transition temperature and poor solubility in common organic solvents. The incorporation of bulky [3, 8] or the use of pendant monomers [3, 9] such as biscoumarin** 1** containing polar carbonyl groups along with introduction of flexible spacer [3, 10, 11] in the polymer chain tends to reduce the interaction between polymer chains and eventually leads to increase in free volume and solubility with improvement in processability [12–15] by maintaining its thermal stability. When considering the properties of biomaterial [16], the first and premier requirement is chemically inert/active, nontoxic, and sustainable by the human body. Implantation of surgical devices like sutures, prosthesis anchor, staples, and valves may lead to incorporation of foreign object into the human body that has the potential to infect patients with various microbes. Sometimes, this surgical implantation leads to harmful side effects like inflammation; if it persists for long time, it ultimately leads to dysplasia. Coumarin and its derivatives are one of the important classes of organic compounds possessing intense biological activity including anticoagulant, estrogenic, antimicrobial, analgesic, antibacterial, antifungal, anti-inflammatory, antitumor, and anti-HIV. Of particular interest coumarin derivative with styrene pharmacophore having trifluoromethyl group at the meta position enhances its biological activity. By compiling the above facts, it is necessary to develop polymer possessing easy fabrication process and fulfil the requirement of biomaterial by showing activities against microbial stains and some cancers. The aim of the present investigation is to synthesize and characterize a random copolyester containing biological active biscoumarin group bearing 3-(trifluoromethyl)styrene by inherent viscosity measurements, solubility tests, FTIR, ^1^H-NMR spectroscopy, X-ray diffraction analysis (XRD), thermogravimetric analysis (TGA), and differential scanning calorimetry (DSC). The synthesized copolyester was subjected to* in vitro* anticancer activity against lung cancer (*Hep-2*) cell line and antimicrobial studies.

## 2. Experimental

### 2.1. Materials and Methods

4-Hydroxycoumarin (Aldrich) was used as such. 3-(trifluoromethyl) Cinnamaldehyde, hydroquinone, azeloyl chloride, dimethylacetamide, and pyridine (Aldrich) were used as received. Solvents were purified according to the standard procedure in the literature [17].* Hep-2 *cell lines were obtained from National Centre for Cell Sciences, Pune (NCCS). The cells were maintained in minimal essential medium (MEM) supplemented with 10% FBS, penicillin (100 *μ*g/mL), and streptomycin (100 *μ*g/mL) in a humidified atmosphere of 50 *μ*g/mL CO_2_ at 37°C. MEM was purchased from Hi Media Laboratories. Fetal bovine serum (FBS) was purchased from Cistron laboratories. Trypsin, methyl thiazolyl diphenyl-tetrazolium bromide (MTT), and dimethyl sulfoxide were purchased from Sisco Research Laboratory Chemicals, Mumbai.

Inherent viscosity was determined at a polymer concentration of 0.5 g/dL in NMP at 30°C using an Ubbelohde suspended level viscometer. 3% (w/v) solutions were taken as a standard for solubility of copolyester in various solvents. FT-IR spectra were recorded on Perkin Elmer 883 spectrophotometer. ^1^H-NMR spectra for monomer and polymer were recorded on a Bruker 400 MHz spectrometer using CDCl_3_ as a solvent. Thermogravimetric analysis (TGA) was performed in nitrogen at 10°C/min with a TGA instrument SDT Q600. Differential scanning calorimetry (DSC) was performed with DSC 200 F3 Maia instrument at a heating rate of 10°C/min under nitrogen atmosphere. Gel permeation chromatography (GPC) analysis was used to determine number average molecular weight (*M*_*n*_) and weight average molecular weight of the polymer (*M*_*w*_) by WATERS 501 equipped with three ultrastyragel columns and a differential refractive index detector using THF as eluent and polystyrene standards were employed for calibration. The flow rate was 1 mL/min. X-Ray diffraction measurements were recorded on a Bruker XRD D8 FOCUS using Cu K*α* radiation source.

Antibacterial activity of random copolyester was determined by disc diffusion method on Muller Hinton agar (MHA) medium and Sabouraud Dextrose agar (SDA) medium, respectively. Both Muller Hinton agar and Sabouraud Dextrose agar media were poured into the petri plate. After the medium was solidified, the inoculums were spread on the solid plates with sterile swab moistened with the bacterial suspension. The discs were placed in MHA and SDA plates and 20 *μ*L of sample concentration was added. Each sample was placed in the disc. The plates were incubated for 24 h, at 37°C. Then, the antimicrobial activity was determined by measuring the diameter of zone of inhibition. Standard antibiotic ampicillin (20 *μ*g/disc) was used as reference. Fresh bacterial cultures of gram negative bacteria, namely,* Escherichia coli* (MTCC-729) and* Salmonella typhi* (MTCC-531), and gram positive bacteria* Bacillus subtilis* (MTCC-2274) and* Staphylococcus aureus* (MTCC-3160) were used for the antibacterial test. Antifungal activity of extracts was determined by disc diffusion method on Sabouraud Dextrose agar medium. Disc diffusion assay (DDA) can also be performed for screening by standard method. Stock cultures were maintained at 4°C on nutrient agar slant. Active cultures for experiments were prepared by transferring a loop full of culture from the stock cultures into the test tubes containing Sabouraud Dextrose broth, which were incubated for 24 h at 37°C.* Aspergillus Niger* and* Aspergillus Flavus* were the fungi which were used for the study.

### 2.2. Synthesis of Monomer **3**

**Scheme 1:**
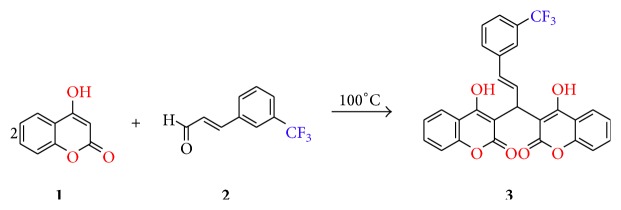
Synthesis of monomer** 3**.

Mixture of 4-hydroxycoumarin** 1** (2 g, 4 mmol) and 3-(trifluoromethyl) cinnamaldehyde** 2** (1.2 g, 2 mmol) was mixed thoroughly and heated in oil bath at 100°C (Scheme 1). After 30 min, the sample was tested by TLC and it was found that there were no monomers present, which indicates the completion of reaction. The reaction mixture was then cooled to room temperature. Hot ethanol (10 mL) was added to the solid mass. The solid product obtained was filtered, dried, and recrystallized using ethanol to give yellow colour powder (2.6 g, 83.8%). *R*_*f*_: 0.25 (*n*-hexane: EtOAc-1 : 1). ^1^H-NMR (400 MHz, CDCl_3_): *δ* (ppm): 11.77 (s, 1H); 11.28 (s, 1H); 8.04–8.02 (t, 2H); 7.63–7.60 (m, 2H); 7.58 (s, 1H); 7.56–7.54 (d, 1H); 7.48–7.47 (d, 1H); 7.43–7.37 (m, 5H); 6.82–6.77 (dd, 1H); 6.58–6.54 (dd, 1H); 5.51–5.50 (m, 1H).

### 2.3. Synthesis of Copolyester **6**

**Scheme 2:**
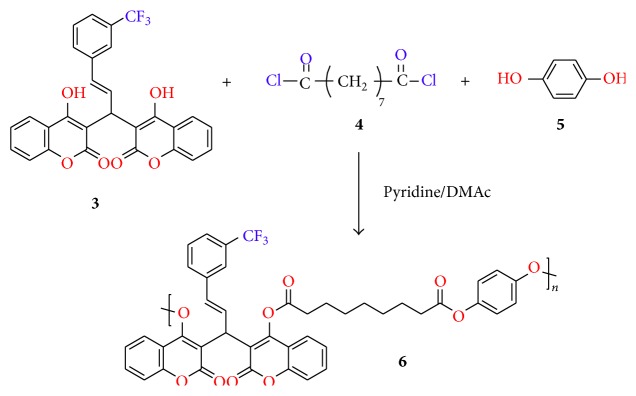
Synthesis of copolyester** 6**.

A three-neck flask fitted with mechanical stirrer was charged with monomer** 3** (0.001 mmol) and hydroquinone** 5 **(0.001 mmol) and fresh distilled DMAc (30 mL) (Scheme 2). The mixture was cooled in an ice bath, 0.004 mmol of pyridine was added, and the temperature was reduced to 0°C. Then, azeloyl dichloride** 4** (0.002 mmol) was added to the suspended reaction mass and the resulting mixture was stirred at room temperature for 1 h and then at 80°C for 7 h. The reaction mixture was poured into 50 mL methanol and the precipitated polymer was collected by filtration, washed thoroughly with methanol, and dried at 80°C under vacuum for 10 h to give pale yellow colour amorphous powder (yield: 71.8%).

### 2.4. *In Vitro* Assay for Anticancer Activity (MTT Assay)

The MTT assay was performed as first described by Mosmann with the modifications suggested by Denizot and Lang. Cells (1 × 10^5^/well) were plated in 24-well plates and incubated at 37°C with 5% CO_2_ condition. After the cell reaches the confluence, the various concentrations of copolyester** 6** were added and incubated for 24 h. After incubation, the sample was removed from the well and washed with phosphate-buffered saline (pH 7.4) or MEM without serum. 100 *μ*L/well (5 mg/mL) of 0.5% 3-(4,5-dimethyl-2-thiazolyl)-2,5-diphenyl-tetrazolium bromide (MTT) was added and incubated for 4 h. After incubation, 1 mL of DMSO was added in all the wells. The absorbance at 570 nm was measured with UV-spectrophotometer using DMSO as the blank. Measurements were performed and the concentration required for a 50% inhibition (IC_50_) was determined graphically. The % cell viability was calculated using the following formula:

(1) $$\begin{array}{c} \%\;\text{cell}\;\text{viability}=\left(\frac{A_{570}\;\text{of}\;\text{treated}\;\text{cells}}{A_{570}\;\text{of}\;\text{control}\;\text{cells}}\right)\times 100. \end{array}$$

Graphs were plotted using the % of cell viability in *y*-axis and concentration of the sample in *x*-axis. Cell control and sample control were included in each assay to compare the full cell viability in cytotoxicity and anticancer activity assessments.

## 3. Results and Discussion

### 3.1. FTIR and ^1^H-NMR Analysis

**Figure 1:**
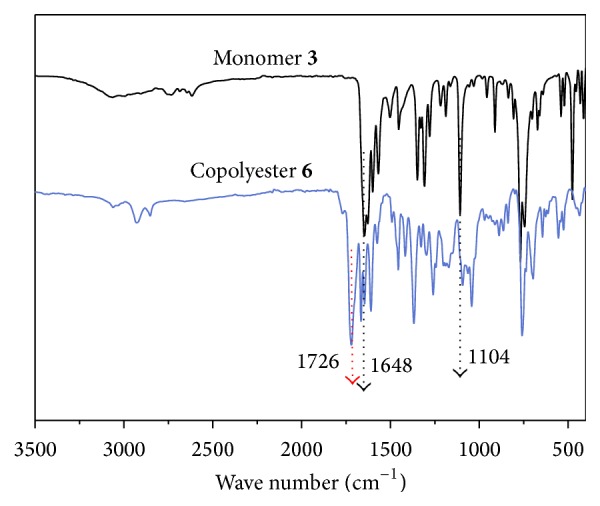
FT-IR spectra of monomer and copolyester.

**Figure 2:**
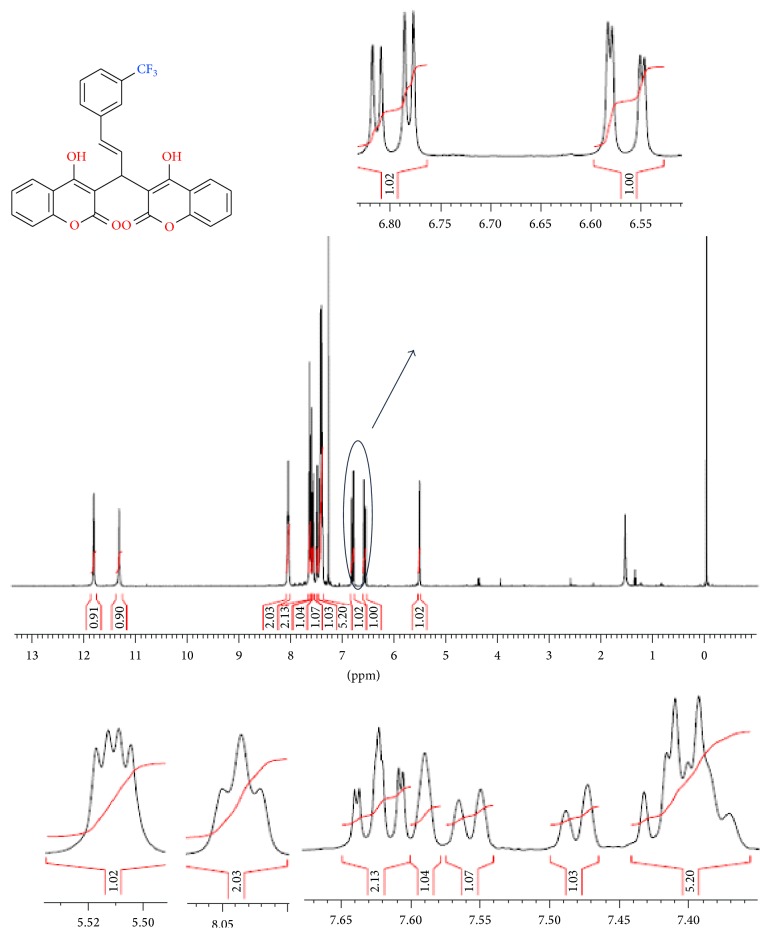
^*1*^ *H-NMR* spectrum of monomer** 3** in CDCl_3_.

**Figure 3:**
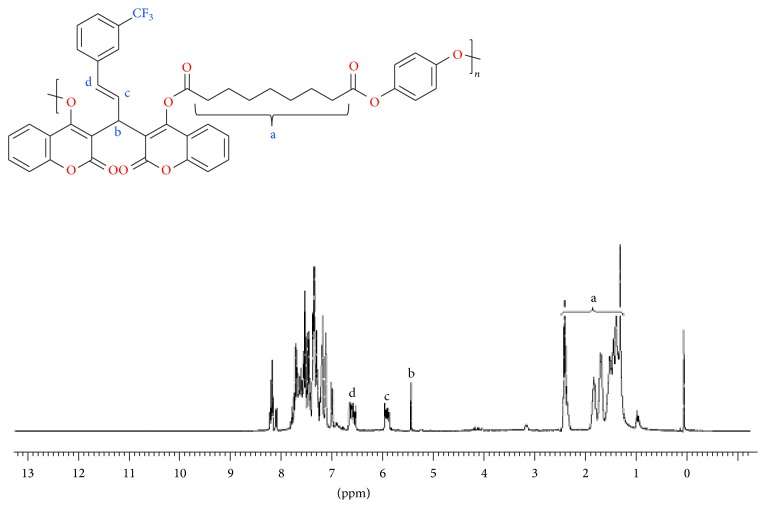
^*1*^ *H-NMR* spectrum of copolyester** 6** in CDCl_3_.

The structures of the copolyester were confirmed using FTIR, ^1^H-NMR spectroscopy, the representative FTIR spectrum of both monomer** 3** and copolyester** 6** was shown in Figure 1. The polymer exhibited characteristic absorption bands at 1726 cm^−1^; this is attributed to the presence of carbonyl stretching of ester group. Both monomer and polymer exhibited peak at 1648 cm^−1^; this is due to the stretching of carbonyl group of biscoumarin unit. The absorption band at 1104 cm^−1^ is due to the stretching vibration of C-F in both monomer** 3** and polymer** 6**. TMS (tetramethylsilane) as internal standard and CDCl_3_ as solvent at room temperature were used to analyze the ^1^H-NMR spectra of monomer** 3** and its respective copolyester** 6**. The ^1^H-NMR spectra of monomer** 3** and copolyester** 6** are presented in Figures 2 and 3. In ^1^H-NMR spectrum of monomer, the two protons of cinnamyl group appeared at 6.82–6.77 ppm and 6.58–6.54 ppm and it has been encircled distinctly in Figure 2. The two hydroxyl groups seem to appear at two different positions 11.77 and 11.28 ppm, representing that these two groups are nonequivalent. In the proton NMR of copolyester, bridged carbon bearing pendant 3-(trifluoromethyl)styrene (peak b) appeared at 5.55 ppm, thus confirming the incorporation of biscoumarin monomer** 3** in the polymeric chain. The characteristic peak at 1.13–2.34 ppm is attributed to the aliphatic acid dichloride. The aromatic protons related to biscoumarin** 3** and hydroquinone** 5** in the polymer backbone appeared in the region of 8.22–7.10 ppm and two peaks in the region of 6.66 and 5.91 ppm related to the alkene protons (–CH=CH–, peak c & d) of the styrene group, respectively.

### 3.2. Polymer Solubility

Solubility parameter is an important criterion which has to be considered for polymer processing. The copolyester showed good solubility in chlorinated solvents and polar aprotic solvents; this may be attributed to the presence of unsymmetric biscoumarin moiety which leads to reduced rigidity of polymer chains. This increase in solubility may be attributed to the incorporation of bulky 3-(trifluoromethyl)styrene pendant unit, which disrupts the polymer chain and hinders close chain from packing there by reducing chain interactions. Thus, it is inferred that the incorporation of 3-(trifluoromethyl)styrene pendant unit influence more in solubility.

### 3.3. Inherent Viscosity

The inherent viscosity of the copolyester was determined in NMP at 30°C at the concentration of 0.5 g/dL using an Ubbelohde suspended level viscometer by calculating the values of flow time of pure solvent and copolyester. The inherent viscosity of the copolyester was found to be 0.15 dL/g. In general, inherent viscosities increase with increase in molecular weight. It is evident that copolyester exhibits lower viscosity value because the presence of unsymmetrical bulky monomer with 3-(trifluoromethyl)styrene pendant unit may tend to reduce the polymer chain length and hence inherent viscosity value decreases. It is also reflected in the GPC data. The number average molecular weight (*M*_*n*_) and weight average molecular weight (*M*_*w*_) of the copolyester** 6** are 4,489 and 6,138 with 1.36 as polydispersity index value, respectively. It is inferred that the copolyester formed in a random manner and lower molecular weight of the polymer is suitable for the biological application.

### 3.4. DSC and TGA Analysis

**Figure 4:**
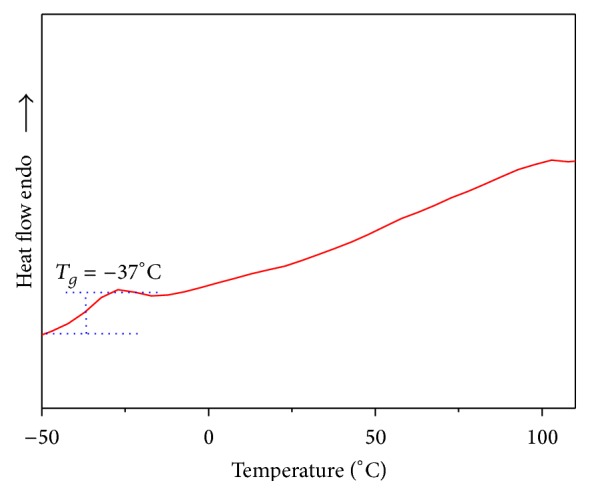
DSC thermogram of random copolyester** 6**.

The *T*_*g*_ of the copolyester was determined by DSC at a heating rate of 10°C min^−1^ under nitrogen atmosphere and the DSC thermograms of copolyester** 6** shows glass transition temperature (*T*_*g*_) at −37°C as presented in Figure 4. The convincing explanation for this low glass transition temperature can be attributed to the fact that the pendant 3-(trifluoromethyl)styrene in the polymeric structure was connected to the biscoumarin unit, which made the polymer chains unable to array regular and hinders the close chain packing, thus reduce the rigidity of the polymeric structure. As expected *T*_*g*_ was dependant on the structure of diacid chloride component. Thus, it is evident that pendant group and flexible spacer decreases rigidity ultimately lead to low glass transition temperature.

**Figure 5:**
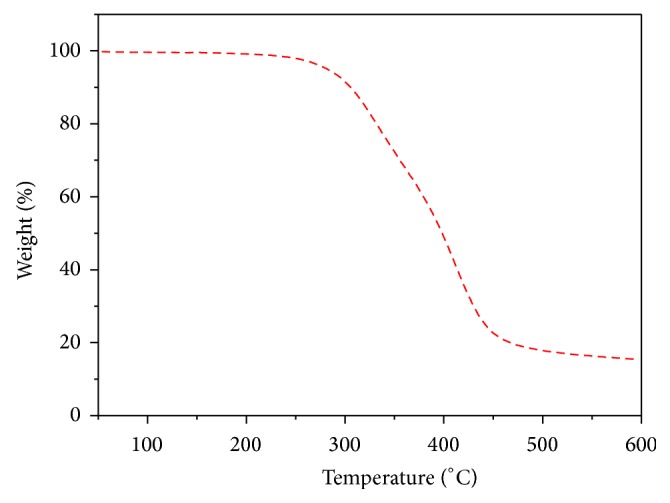
TGA curve of copolyester** 6**.

The influence of polymer structure on the thermal properties of copolyester was investigated by thermogravimetric analysis (TGA) at a heating rate of 10°C min^−1^ in nitrogen atmosphere and the TGA curve was shown in Figure 5. The initial decomposition temperature (IDT), the temperature for 10% weight loss (*T*_10_), and the maximum decomposition temperature (*T*_max⁡_) of copolyesters are 251°C, 305°C, and 397°C. Initial decomposition temperature and the temperature at 10 w% loss (*T*_10_) indicate its good thermal stability.

### 3.5. XRD Analysis

**Figure 6:**
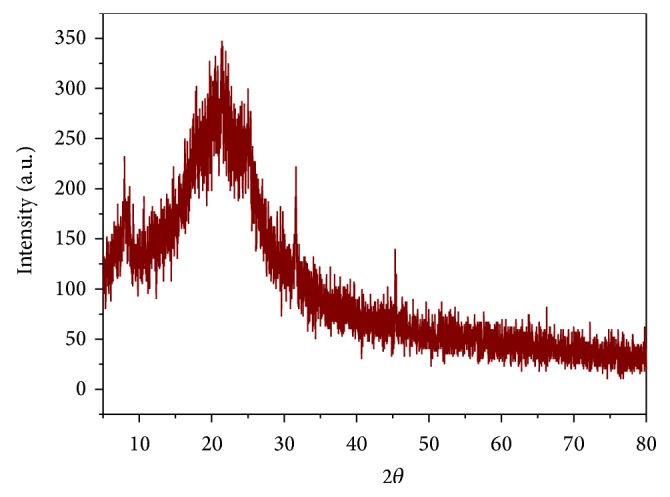
X-ray diffractogram of copolyester** 6**.

X-ray diffraction patterns of copolyester** 6** exhibit the essential amorphous nature of this random copolyester. Copolyester exhibits some diffused degree of crystallinity at 2*θ* range of about 7°, 31°, and 45°. This observation could be explained that the introduction of flexible spacer is responsible for the least degree of crystallinity shown in X-ray diffraction pattern. In addition, the presence of pendant 3-(trifluoromethyl)styrene reduced coplanarity, hindered the dense packing of polymer chains, and destroyed the ordered arrangement, resulting in amorphous nature of these polyesters, which is also reflected in their improved solubility. Copolyester** 6** exhibits with conspicuous amorphous halo at 2*θ* range of about 15° to 30° as shown in Figure 6.

### 3.6. Antibacterial and Antifungal Studies

**Figure 7:**
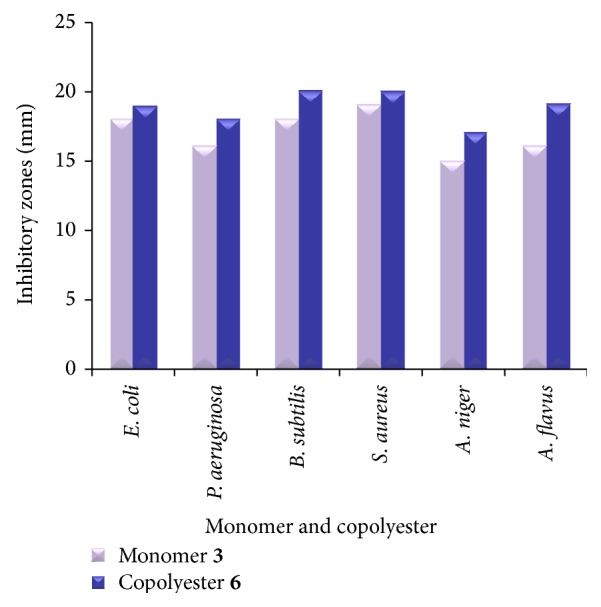
Comparative activities of monomer** 3** and copolyester** 6** against various microbes.

The purpose of antibacterial studies was to find out the efficacy of copolyester** 6** to inhibit the growth of microorganisms in the near vicinity of surgical implantation. The antibacterial activities of the synthesized monomer** 3 **[18, 19] and the copolyester** 6 **were tested against gram positive and gram negative bacteria, and their results for the antibacterial zone they inhibit are presented in Table 1. The comparative activities of monomer** 3** and copolyester** 6** against microbes are shown in Figure 7. Copolyester** 6** with pendant 3-(trifluoromethyl)styrene exhibited moderate to good activity against gram positive and gram negative bacteria compared with monomer** 3**; this may be due to the enhanced lipophilicity character of the copolyester. From the data of antifungal activity, it is observed that the compounds are highly active against* A. flavus* compared with* A. niger* which exhibits moderate activity as shown in Table 2. The cell wall contains many phosphates, carbonyl and cysteinyl ligands which maintain the integrity of the membrane by acting as a diffusion barrier. Furthermore, increased lipophilicity enhances the penetration of the copolyester into lipid membrane and disturbs the respiration process of the cell and thus blocks the synthesis of the proteins which restricts further growth of the organism.

The results towards these microbes revealed that the incorporation of pendant 3-(trifluoromethyl)styrene into the biscoumarin monomer** 3**, of particular that the presence of trifluoromethyl group at the meta position of styrene enhanced its activity, apart from this the polymerised product still enhanced its overall activity due to the lipophilic character.

### 3.7. Anticancer Evaluation of Copolyester

**Figure 8:**
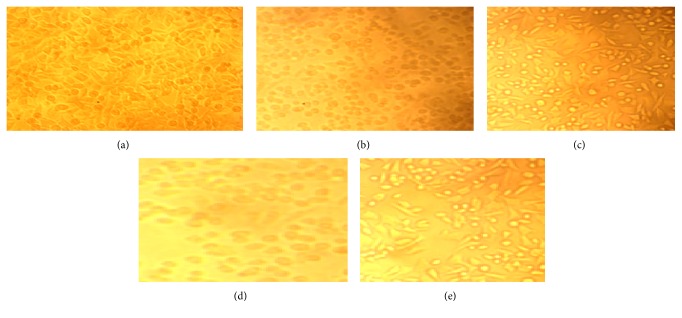
Anticancer evaluation of copolyester** 6** on* Hep-2* cell line at various concentrations: (a) normal* Hep-2* cell line, (b) 1000 *μ*g/mL, (c) 250 *μ*g/mL, (d) 125 *μ*g/mL, and (e) 62.5 *μ*g/mL.

**Figure 9:**
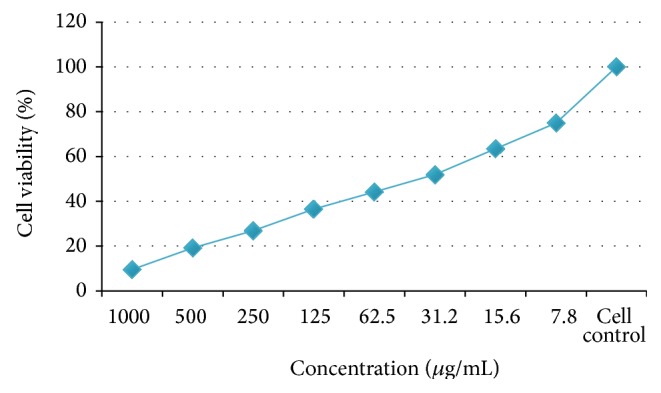
Graphical representation of activities of copolyester** 6** in the MTT Assay.

Viable cells were determined by the absorbance. Concentration required for a 50% inhibition of viability (IC_50_) was determined graphically from Figure 9. The absorbance was measured with a UV-spectrophotometer using wells without sample containing cells as blanks. The effect of the copolyester** 6** on the proliferation of* Hep-2* cell line was expressed as the % cell viability. The affected* Hep-2* cell line at different concentration was shown in Figure 8. The covalent conjugation of biscoumarin bearing pendant 3-(trifluoromethyl)styrene through ester linkage increases its molecular size and steric hindrance may improve its cellular permeability and restricts its cancer progression [20, 21]. It is explicit from Table 3 that low concentration of copolyester induced greater anticarcinogenic activity effects on* Hep-2* cell line than the individual agents.

## 4. Conclusion

In summary, the present study allows the conclusions that the copolyester showed good solubility in various organic solvents and it is explicit from the DSC data that the copolyester exhibits low glass transition temperature by maintaining its thermal stability; this is attributed to the incorporation of pendant 3-(trifluoromethyl)styrene group in the biscoumarin unit. The structures of the random copolyester were confirmed by FT-IR and NMR spectroscopy. And incorporation of pendant 3-(trifluoromethyl)styrene decreases the percentage of crystallinity which confirms the tendencies towards amorphous nature of copolyester exhibited by X-ray diffraction measurements. The cytotoxic assay shows that the copolyester is toxic to the* Hep-2* cell line in lower concentration. It is explicit from the antimicrobial data that the copolyester exhibits significant biological activity against* Escherichia coli*,* salmonella typhi*,* Bacillus subtilis*, and* Staphylococcus aureus*. These lines of evidence show that the copolyester can be preferred as biomaterial in various biomedical applications.

## Figures and Tables

**Table 1:** Zones of inhibition (in mm) of the compounds against various bacteria.

Compound	*E. coli *			*P. aeruginosa *			*B. subtilis *			*S. aureus *		
	20 *μ*g	40 *μ*g	60 *μ*L	20 *μ*g	40 *μ*g	60 *μ*g	20 *μ*g	40 *μ*g	60 *μ*g	20 *μ*g	40 *μ*g	60 *μ*g
Monomer **3**	16	16	18	15	16	16	17	17	18	16	16	19
Copolyester **6**	18	18	19	17	18	18	18	19	20	19	19	20

**Table 2:** Zones of inhibition (in mm) of the compounds against fungus.

Compound	*A*. *niger *			*A*. *flavus *		
	20 *μ*g	40 *μ*g	60 *μ*g	20 *μ*g	40 *μ*g	60 *μ*g
Monomer **3**	14	14	15	15	16	17
Copolyester **6**	15	15	16	17	18	19

**Table 3:** Anticancer activities of copolyester **6** on *Hep-2* cell line.

Serial number	Concentration (*μ*g/mL)	Dilution	Absorbance (OD)	Cell viability (%)
1	1000	Neat	0.05	9.61
2	500	1 : 1	0.10	19.23
3	250	1 : 2	0.14	26.92
4	125	1 : 4	0.19	36.53
5	62.5	1 : 8	0.23	44.23
6	31.2	1 : 16	0.27	**51.92**
7	15.6	1 : 32	0.33	63.46
8	7.8	1 : 64	0.39	75.00
9	Cell control	—	0.52	100
